# Validity Analysis of Monocular Human Pose Estimation Models Interfaced with a Mobile Application for Assessing Upper Limb Range of Motion

**DOI:** 10.3390/s24247983

**Published:** 2024-12-14

**Authors:** Rayele Moreira, Silmar Teixeira, Renan Fialho, Aline Miranda, Lucas Daniel Batista Lima, Maria Beatriz Carvalho, Ana Beatriz Alves, Victor Hugo Vale Bastos, Ariel Soares Teles

**Affiliations:** 1 Postgraduate Program in Biotechnology, Parnaíba Delta Federal University, Parnaíba 64202-020, Brazil; moreirarayele@gmail.com (R.M.);; 2 University Center Inta, Sobral 62050-100, Brazil; 3 Campus Araioses, Federal Institute of Maranhão, Araioses 65570-000, Brazil

**Keywords:** range of motion, upper limbs, goniometry, human pose estimation, computer vision

## Abstract

Human Pose Estimation (HPE) is a computer vision application that utilizes deep learning techniques to precisely locate Key Joint Points (KJPs), enabling the accurate description of a person’s pose. HPE models can be extended to facilitate Range of Motion (ROM) assessment by leveraging patient photographs. This study aims to evaluate and compare the performance of HPE models for assessing upper limbs ROM. A physiotherapist evaluated the degrees of ROM in shoulders (flexion, extension, and abduction) and elbows (flexion and extension) for fifty-two participants using both Universal Goniometer (UG) and five HPE models. Participants were instructed to repeat each movement three times to obtain measurements with the UG, then positioned while photos were captured using the *NLMeasurer* mobile application. The paired *t*-test, bias, and error measures were employed to evaluate the difference and agreement between measurement methods. Results indicated that the *MoveNet Thunder* INT16 model exhibited superior performance. Root Mean Square Errors obtained through this model were <10° in 8 of 10 analyzed movements. HPE models demonstrated better performance in shoulder flexion and abduction movements while exhibiting unsatisfactory performance in elbow flexion. Challenges such as image perspective distortion, environmental lighting conditions, images in monocular view, and complications in the pose may influence the models’ performance. Nevertheless, HPE models show promise in identifying KJPs and facilitating ROM measurements, potentially enhancing convenience and efficiency in assessments. However, their current accuracy for this application is unsatisfactory, highlighting the need for caution when considering automated upper limb ROM measurement with them. The implementation of these models in clinical practice does not diminish the crucial role of examiners in carefully inspecting images and making adjustments to ensure measurement reliability.

## 1. Introduction

Human Pose Estimation (HPE) is a computer vision (CV) task employed for the identification of key points on the human body [1], enabling the description and tracking of an individual’s pose [2]. This field has gained popularity in computer vision, so finding applications in practical domains such as user interface security, motion capture for filmmaking, and medical applications [3,4]. The progress in computer vision and artificial intelligence has significantly streamlined the process of automatic medical imaging analysis, playing a vital role in modern medicine [5]. In the medical realm, HPE models have been utilized to track general motor development, optimize human performance, prevent injuries, ensure safety, and conduct motor and postural assessments [6,7].

HPE models have been successfully integrated into smartphone applications, leveraging the widespread use of these devices in medical practice. The rising number of smartphone applications designed for physical assessment in healthcare capitalizes on the capabilities of integrated cameras and sensors, including accelerometers and gyroscopes. Physical examinations, such as posture evaluation [8] and gait analysis [9], can now be conveniently conducted using smartphones. Moreover, certain mobile applications dedicated to Range of Motion (ROM) assessment have been developed and validated [10,11].

ROM assessment constitutes a pivotal aspect of physical evaluation, enabling the identification of joint and muscle limitations while serving as a metric to track the patient’s rehabilitation progress. Traditional ROM assessment involves the use of a Universal Goniometer (UG), which is positioned on the joint of interest, employing manually identified Key Joint Points (KJPs) as reference points [12]. Alternatively, sensor-based mobile applications [13] facilitate measurements by placing the device directly over the joint (i.e., axis of motion) or along the body segment (e.g., arm, leg), and the sensor detects the angular displacement of the segment during movement [14]. Also, Organic Field-Effect Transistor (OFET) sensors have been introduced as tools for monitoring human motion, such as arm movement [15]. However, measurements obtained through such sensor-based tools may be susceptible to inaccuracies due to human error in identifying KJPs and challenges in securely attaching the device to the patient’s limb, potentially leading to device displacement.

In image-based applications, a photo of the body segment or radiograph of the segment is captured and a protractor or Virtual Goniometer (VG) is used to measure the Cobb angle or the joint angle. Examples of such applications include *Cobbmeter* [16] and *iPinPoint* [17,18]. However, these applications leverage the acceleration capabilities of smartphones, and even when using computer vision techniques, they are not fully automated. This means that the examiner still manually positions the points and drags them on the smartphone screen.

Deep learning/machine models capable of estimating human pose have not been originally designed for clinical settings. However, they enable the automatic identification of KJPs used as references in human physical assessments. As a result, these technologies have been applied across different clinical contexts, including physical and kinematic assessments [6,19]. Despite the growing use of HPE models for human physical assessments, these models rely on unconstrained images, meaning there are no standardized protocols for the image collection used to train them. While HPE models are extensively utilized in different fields (e.g., games, robotics, 3D animation of human actions, sports) [20,21,22], these applications are generally broad in scope, and they are designed to detect the human body without focusing on the accuracy in measurements. Also, it is important to note that such models have not been specifically developed for biomedical and biomechanical applications, such as goniometry used in healthcare. Therefore, it is necessary to evaluate and compare the performance of HPE models for specific tasks, such as goniometric assessments, to determine their reliability and which present better performance for effective clinical application.

Based on this research gap, a few studies have addressed the performance evaluation of HPE models for human ROM assessment. Fan et al. [23] evaluated the reliability of *OpenPose* in assessing upper limb ROM compared to evaluations conducted by human assessors. On the other hand, Sabo et al. [24] applied different models for video-based ROM analysis and compared them with a digital goniometer. Different from the previous works, we extend our initial studies [7,19,25] by applying HPE models to measure human upper limb ROM, based on the analysis of body images captured using the *NLMeasurer* mobile application [19,25]. Our study in [19] theorizes the potential use of HPE models in biomedical applications, such as for assessing posture and ROM. In contrast, our studies in [7,25] aimed to experimentally validate HPE models, focusing exclusively on assessing posture and anthropometry.

The objective of this study is to evaluate and compare the performance of different HPE models (*PoseNet* and four variations of *MoveNet*) integrated into a smartphone application for ROM assessment of the human upper limbs in monocular view [26]. We restricted the movements under evaluation to focus on the gross movements of the shoulder (flexion, extension, and abduction) and elbow (flexion and extension) joints. These joints play a crucial role in providing humans with functional capabilities, providing ROM that can extend up to 180 degrees for the shoulder and 145 degrees for the elbow. This extensive mobility is critical for effectively performing a diverse range of human motor tasks.

## 2. Materials and Methods

### 2.1. Human Pose Estimation for ROM Assessment

HPE models experimented in this study were [27] *MoveNet Lightning* (FP16 and INT8 quantized), *MoveNet Thunder* (FP16 and INT8 quantized), both in version 4 using *MobileNetV2* architecture and provided in *TensorFlow Lite* (Python language), and *PoseNet* [28] running on the *TensorFlow.js* [29] library working with *MobileNet* architecture. They are open-source models [30,31] that use Convolutional Neural Networks (CNNs) to identify the position of specific joints in the human body from images or videos. Their structure is optimized to identify key points of the body such as shoulders and elbows. While *PoseNet* uses the MobileNet architecture, *MoveNet Lightning* and *Thunder* use MobileNetV2, which is known for its balance between efficiency and accuracy. MobileNetV2 is designed for resource-constrained devices, using separable convolutions to reduce computational complexity. *MoveNet Lightning* is smaller and faster, but less accurate than the *Thunder* version.

Each point detected by models is named *keypoint* and contains a pair of coordinates *x* and *y*, a value varying between 0.0 and 1.0, which represents confidence scores associated with each *keypoint* inference made by the model and a description of the member identified. The model can be operated to identify a single pose or multiple poses. In this study, only single-pose mode was used, since the goniometric evaluation is performed separately for each patient according to the body segment to be assessed.

**Figure 1:**
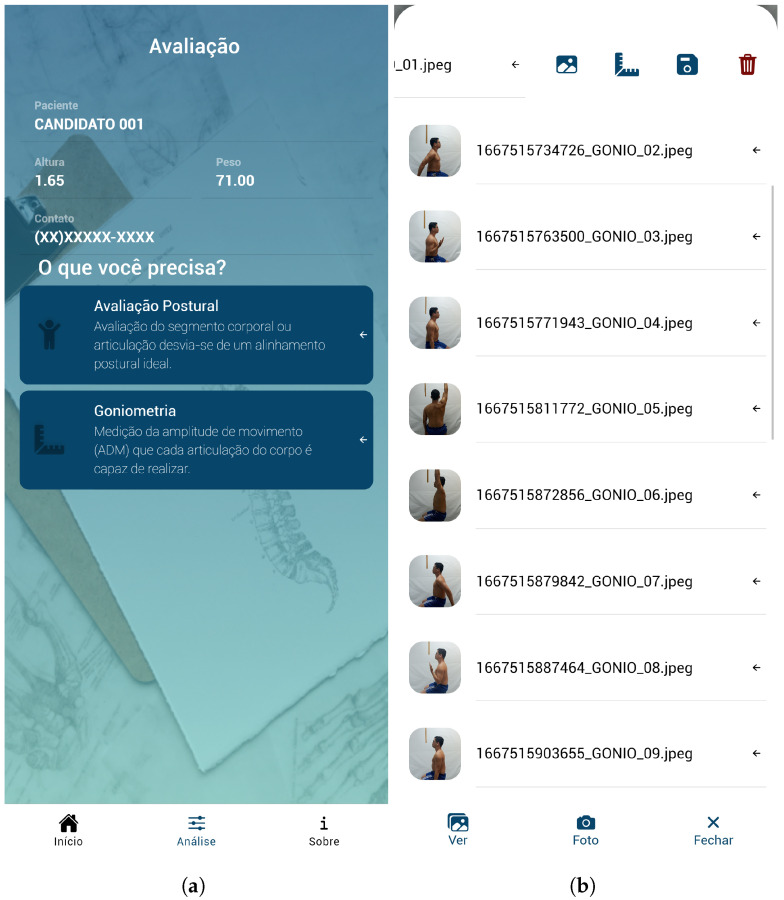
*NLMeasurer* screenshots (texts in Brazilian Portuguese (PT-BR) language) with application screen with (**a**) participant records to start an assessment and buttons with two types of assessment (postural and goniometry); and (**b**) a list of all captured images of the one participant.

We evaluate the performance of the HPE models to measure the ROM of shoulder and elbow movements in monocular view. Photos are taken using an image-based application proposed for human physical assessment [7,19,25]: the examiner registers participant information, selects the goniometry option (Figure 1a), and takes pictures. The examiner collects images of all movements in high definition (1440 × 720), so creating a list of images to be assessed (Figure 1b). All images are sent to the cloud for further processing by the models, which automatically return KJPs and the degrees in the joint movement analyzed.

**Figure 2:**
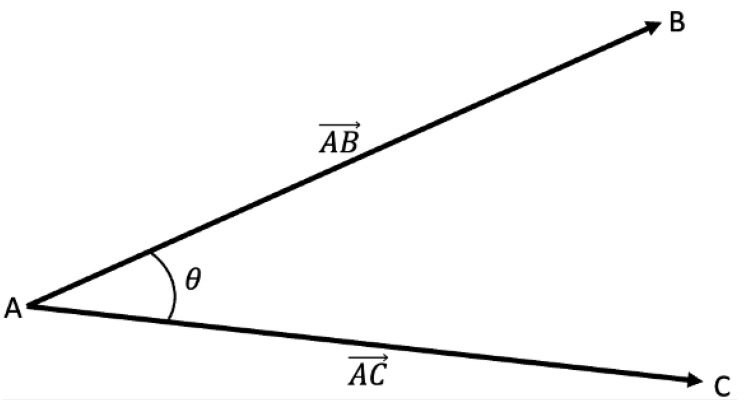
Angle between two body segments.

The angle between two segments, as shown in Figure 2, is calculated based on the scalar product of two vectors, which is equal to multiplying the magnitude of the vectors by the cosine of the angle formed between them. Therefore, the angle Θ can be obtained from Equation (1) [32].

(1) $$\Theta =ArcCos\frac{\vec{AB}\times \vec{AC}}{\left|\vec{AB}\right|\left|\vec{AC}\right|}$$

**Figure 3:**
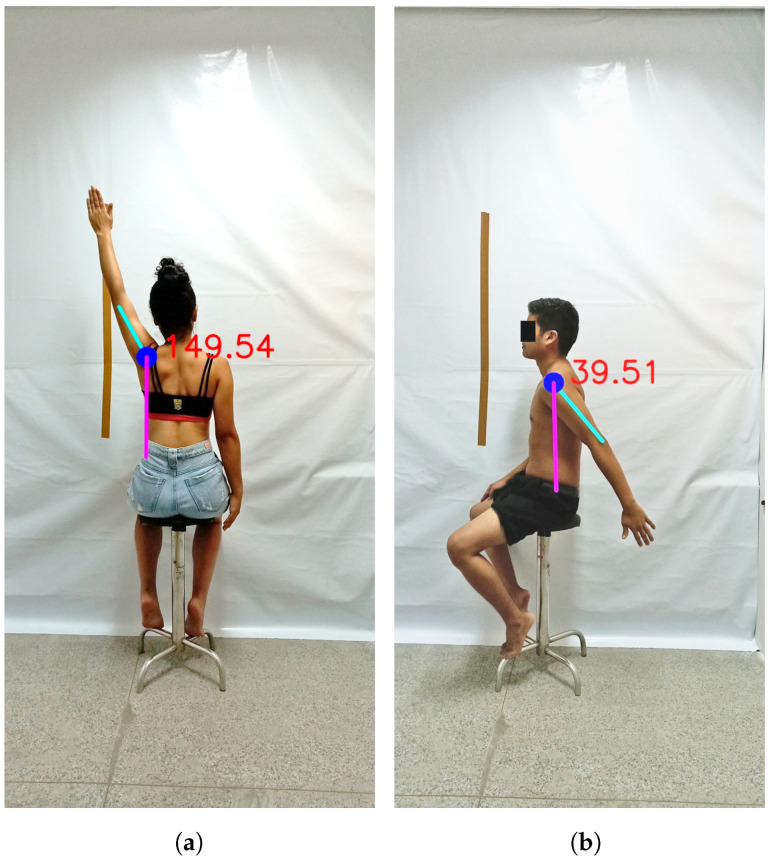
Virtual goniometer showing degrees (i.e., values for ROM) drawn on the device screen: (**a**) VG in left shoulder abduction; and (**b**) VG in left shoulder extension.

For reproducibility purposes, the full code with HPE models used to identify *keypoints* and assess movements is available on GitHub (https://github.com/lucasdblucas/NLMeasurer-Goniometry (accessed on 10 November 2024)). The result generated is an image (Figure 3) with a virtual goniometer drawn over the assessed joint segment and the angle of the movement performed.

### 2.2. Sample

The participants were recruited at the Parnaíba Delta Federal University, Brazil. The selection criteria were the following: healthy subjects, without a history of vestibular or orthopedic diseases that compromise balance and standing, and without other physical or psychiatric pathologies that compromise the understanding and performance of the experimental procedure. Subjects who refused to be photographed according to the experimental protocol were excluded. Ethical approval was obtained from the institutional review board of the Parnaíba Delta Federal University (number 5.876.385). The purpose, nature, procedure, and potential risks of the experiment were fully explained to the participants, and all of them provided written informed consent before participating in the study.

### 2.3. Data Acquisition Procedures

Initially, demographic information including age, gender, weight, and height was collected from each participant. Subsequently, participants were instructed to sit on a chair with a height of 55 cm and perform the following movements: (1) shoulder flexion, extension (lateral view), and abduction (posterior view); (2) elbow flexion and extension (lateral view). Each movement was repeated three times for each limb (right and left arms) and measured using a UG tool by a physiotherapist (i.e., the examiner). By following established literature protocols involving UG, each measurement was repeated thrice, and the mean value was computed for analysis [33,34]. The UG was used as a comparative reference because it is widely accepted in clinical settings for ROM assessment and offers excellent strength of agreement (ICC ≥ 0.83) [35] and sufficient accuracy when compared with radiographic measurements [36]. The recorded ROM degrees for each joint were documented in spreadsheets for subsequent analysis.

Afterward, each participant was positioned in front of a white backdrop measuring 2 m × 2.80 m and instructed to repeat each movement with their right and left arms and, upon reaching the final ROM, maintain a stationary position while the photos were taken using the *NLMeasurer*. Despite the intricate nature of human movement, ROM measurements are standardized and conducted separately for each joint. This principle applies uniformly when assessing both sides of the body. Each joint has its unique average ROM and, even for healthy individuals, variations between the right and left sides may exist. The examiner held the Redmi Note 10S smartphone (Manufacturer: Xiaomi; Sourced in Parnaíba, Brazil; Specifications: Android 10, MediaTek Helio G95 processor-2050 MHz Octa-Core, 128 GB storage, 6 GB RAM, and 64 MP camera) and positioned herself 2.5 m away from the participants for data collection. The application was equipped with an orientation system to ensure image capture only when the device was adequately aligned. Captured photos were stored for subsequent analysis. During data collection employing both the UG and *NLMeasurer*, participants were not required to wear standardized clothing. However, male participants were instructed not to wear shirts, while female ones were instructed to wear sleeveless t-shirts, and participnts of both genders were advised not to wear long pants. Additionally, no specialized lighting system was employed. The decision to forgo specific clothing and a special lighting system was deliberate, aiming to replicate the environment and conditions commonly encountered in clinical settings. Next, all images underwent analysis using a processing pipeline that automatically provided KJPs and degrees of joint movement, as detailed in Section 2.1.

To assess the models’ performance, each movement for either side of the body was treated as a distinct entity. In other words, the flexion of the right shoulder constituted one movement, while the flexion of the left shoulder represented another. This approach was consistently applied to the remaining joints, resulting in a total of 10 distinct movements being evaluated.

### 2.4. Statistical Analysis Procedures

The statistical procedure was applied to the data automatically generated by the models, i.e., no manual adjustment was made to the positioning of the joint points detected by them. Expectation Maximization was applied to handle missing data [37]. Bootstrapping procedures (1000 re-samples; 95% CI BCa) were performed to obtain greater reliability of the results, to correct deviations from the normality of the sample distribution, and also to present a 95% confidence interval for the differences between the averages [38]. The paired *t*-test was used as a parametric analysis of variance to identify any differences between UG and Models. Additionally, Bland–Altman analysis [39] was used to characterize the agreement between each model and UG through the calculation of the mean difference (i.e., bias) and 95% Limits of Agreements (LOAs). Pearson correlation was performed to analyze the data heteroscedasticity. Results with *p* < 0.05 were considered statistically significant, leading to the rejection of the null hypothesis and the acceptance that the methods differ from each other. The Root Mean Square Error (RMSE) was measured as an overall measure of consistency. An RMSE of 5° to 10° was considered good, while an RMSE < 5° was excellent [40,41]. The IBM SPSS 29.0 (IBM Corp., Armonk, NY, USA) software package was used in statistical analysis.

## 3. Results

ROM measurements from 52 participants (31 women) were collected. The participants aged between 18 and 47 years (22.9 ± 6.33), weighing between 42.7 kg and 103 kg (63.6 ± 13), and reaching in height between 1.51 m and 1.85 m (1.66 ± 0.09).

Results demonstrate that MoveNet Thunder INT8 quantized (*MNT8Q*) was the model in which the measured values did not differ significantly (*p* > 0.05) from those measured with the UG in 7 of the 10 movements (Table 1 and Table 2). MoveNet Thunder FP16 quantized (*MNT16Q*) model had the second-best performance (*p* > 0.05 in 6 of the 10 movements), followed by the MoveNet Lightning FP16 quantized (*MNL16Q*) (*p* > 0.05 in 6 of the 10 movements) and MoveNet Lightning INT8 quantized (*MNL8Q*) (*p* > 0.05 in 5 of the 10 movements). The *Posenet* model had the worst performance among the models evaluated (*p* > 0.05 in 4 of the 10 movements).

For all upper-body movements investigated, the bias between the models *MNT8Q* and *MNT16Q*, when compared with UG, ranged from 0.52 to 5.05 and 0.35 to 3.75, respectively. Although both models presented low bias (<5°), the agreement values showed that *MNT16Q* presented lower LOA variability (<10°), except for the elbow flexion movement, which errors were 27.39 and 25.27 for right and left sides, respectively.

**Figure 4:**
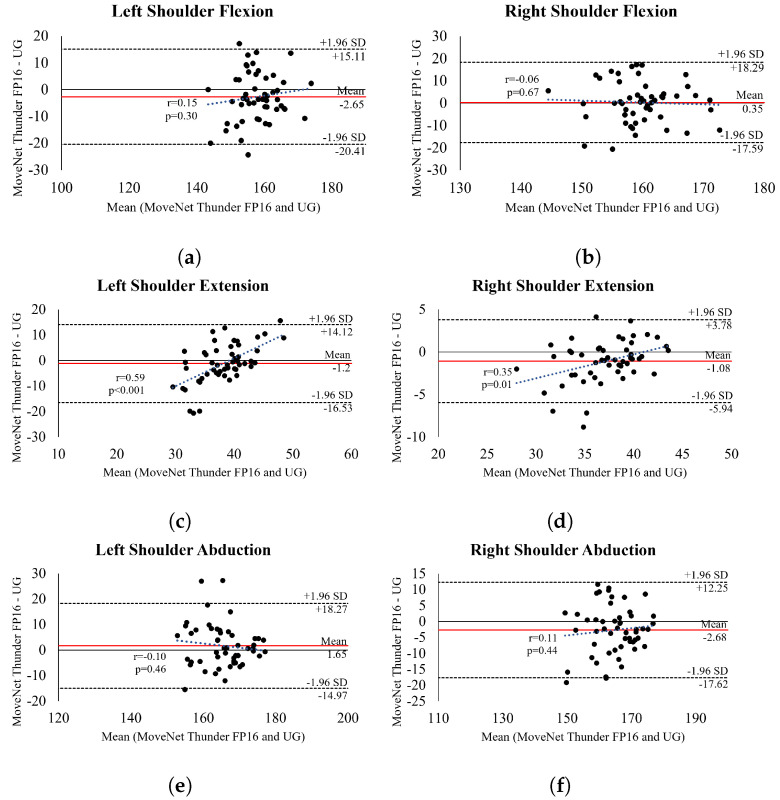
Bland Altman plot showing the level of agreement between *MNT16Q* and *UG* when assessing shoulders. The centered red line shows bias, and the two outer dotted lines represent the upper and lower 95% confidence intervals. The trend line illustrates the correlation between the mean and the difference between the model and UG, serving as a parameter to analyze heteroscedasticity.

**Figure 5:**
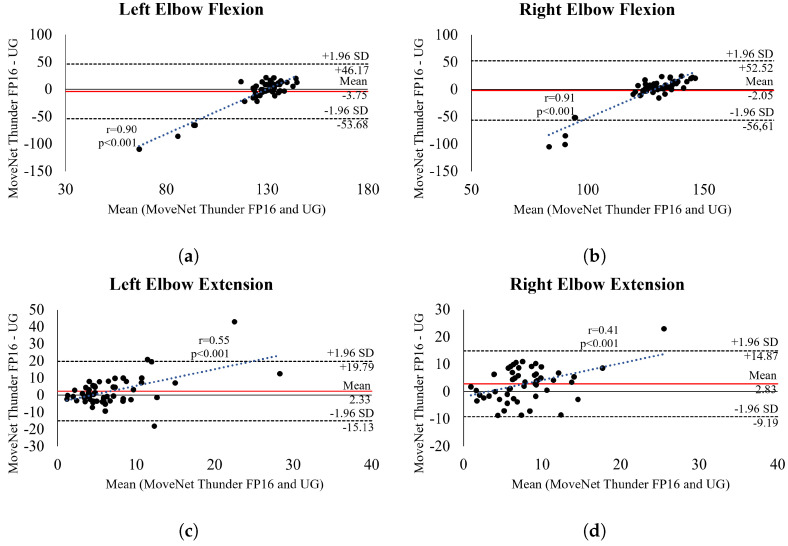
Bland Altman plot showing the level of agreement between *MNT16Q* and UG when assessing elbows. The centered red line shows bias, and the two outer dotted lines represent the upper and lower 95% confidence intervals.The trend line illustrates the correlation between the mean and the difference between the model and UG, serving as a parameter to analyze heteroscedasticity.

Bland Altman plots for the *MNT16Q* (i.e., the best-performing model with lower LOA variability (<10°) and RMSE < 10°) (Figure 4 and Figure 5) also demonstrate good agreement between methods, i.e., most points are within the 95% LOA for all ROM measurements. There was no evidence of heteroscedasticity in the data for shoulder flexion (Figure 4a,b) and abduction (Figure 4e,f) movements, indicating that the HPE model did not consistently err towards overestimation or underestimation. However, for left-sided extension (Figure 4c), there was a trend towards underestimation and overestimation (r = 0.59, *p* < 0.001), whereas for right-sided extension (Figure 4d), there was a trend towards overestimation (r = 0.35, *p* = 0.01). In addition, the analysis of elbow flexion and extension movements revealed a trend for underestimation in left and right flexion (r = 0.90, *p* < 0.001 and r = 0.91, *p* < 0.001) and overestimation in left and right extension (r = 0.55, *p* < 0.001 and r = 0.41, *p* < 0.001). This suggests that the HPE model tended to underestimate elbow flexion angles and overestimate extension angles. It is important to note that such trends were observed due to outliers contributing to the results, as depicted in the graphical distribution. Bland Altman plots for the remaining models are available in Supplementary Material S1.

The relatively large errors were found in elbow flexion also in the *MNT8Q*, which presented errors > 10° in the flexion shoulder left, abduction shoulder left, and extension elbow right. All other models presented large errors, reaching up to 75.8°, as occurred with *PoseNet* (Table 3 and Table 4).

## 4. Discussion

### 4.1. Principal Findings

In this study, we analyzed the performance of five HPE models for upper limbs ROM assessment. Models were trained with varied images, hyperparameters, and architectures of deep artificial neural networks [27,28], but *MNT16Q* and *MNT8Q* exhibited lower RMSE and bias when compared to UG in assessing upper limb ROM. All the models had unsatisfactory performance for elbow flexion. Detecting KJPs appeared to be more challenging during elbow flexion, possibly attributed to the final position of the arm, where the wrist and shoulder are close. We guess that this positioning (i.e., approximation between the wrist and shoulder) may represent what Dubey [3] referred to as complications in poses (i.e., challenges that complicate the accurate detection of human pose), which could have adversely affected the models’ ability to discern and accurately identify KJPs.

**Figure 6:**
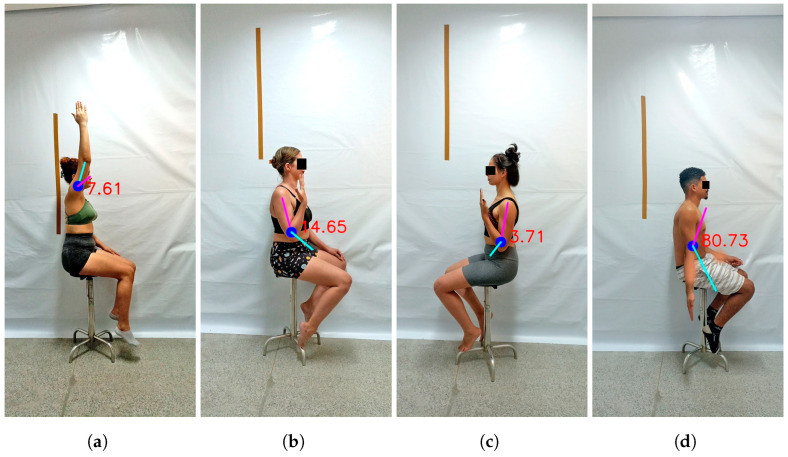
ROM measurements significantly deviating from those expected for healthy individuals by: *MNL8Q* (**a**)—right shoulder flexion and *MNT8Q*; (**b**)—right elbow flexion; (**c**)—left elbow flexion and (**d**)—right elbow extension).

This guess, related to complications in pose, can be only partially supported by the results. Data analysis demonstrated that some values were far from the average value of the ROM expected for each joint. This was evident in the case of elbow flexion, where the ROMs measured were, for example, 14.65 and 3.71 (Figure 6b,c), whereas the typical ROM for healthy individuals is estimated to be around 145 degrees. This is also evident in the Bland-Altman plots (Figure 5), where most measurements of elbow flexion clustered around the mean, yet a few values deviated excessively, thereby impacting the overall outcome. However, similar deviations were also observed in other movements, such as shoulder flexion (Figure 6a) and elbow extension (Figure 6d). Nonetheless, in these cases, deviations were not substantial enough to compromise the performance of the models. While we guess that complications in pose may contribute to these results, it is challenging to pinpoint the exact variable responsible for these data, nor can we attribute it to a single element alone. The specific variable contributing to these outliers remains unclear, but their presence highlights the necessity to develop strategies for enabling models to handle outliers effectively.

Regardless of the contributing variable, outliers are directly associated with the unsatisfactory results of the models, particularly in elbow flexion. Researchers have previously suggested that HPE models demonstrate better robustness when applied to images captured from frontal or posterior views [42]. Our findings reinforce this by showing good results for shoulder flexion and abduction movements, and poorer results for elbow flexion, a movement captured in the lateral view, particularly because all the models showed a wide standard deviation and LOA. Even *MNT16Q*, which demonstrated good agreement with UG also had wide standard deviations and errors > 10°, indicating substantial variation of data around the mean for elbow flexion (Table 2).

HPE models have an average performance in detecting images and videos, but they can face challenges such as inappropriate camera positions that change image perspective [7], especially because the image was captured with the smartphone in the examiner’s hand. Furthermore, participants had different heights and, to frame them in camera, the examiner needed to adjust the smartphone’s position. Adjustments may have produced small oscillations that might change the perspective of the image and, hence, change the model performance. Changes in the perspective can also explain instances where the model failed to accurately identify KJPs, resulting in their positioning outside the participant’s silhouette. Utilizing 3D HPE or optimizing HPE models to better account for perspective variations may reduce errors in KPJs detection and, consequently, in ROM measurements. While there are potential limitations associated with using smartphones, we chose this tool for image capture because it is a more accessible device for professionals in clinical settings.

Types of clothing, lighting, and self-occlusion are pointed out as elements that can impact the performance of the models to detect KJPs, which are used to assess human movement [6]. In this study, participants were not wearing clothing that covered their upper limbs, so the type of clothing does not appear to be a factor that negatively impacted the results. General-purpose HPE models are frequently trained with images of people wearing clothes of different colors and shapes and in different positions and environments. However, for use in clinical settings, standardization of such variables may make HPE models even more accurate to be used in ROM assessment (i.e., a task-specific model). For the general task of tracking the human body, small variations in the detection of the KJPs may not have a big impact, but, to measure human ROM, small variations in the positioning of KJPs can negatively impact angular measurements, and small variations in a ROM can have high clinical significance [43]. Therefore, if a model were tuned incorporating new images to become a task-specific model for goniometry tasks, it could be more likely to become more accurate for measuring ROM and, hence, used with greater reliability in clinical settings. Such a dataset should include images or videos of individuals in relevant positions (e.g., sitting or standing with the limb in initial and final ROM positions) and encompass a diverse range of patients with varying conditions and limitations. This approach to making the model task-specific could equip it to generalize across different clinical scenarios, making it useful for more accurate measurements, and paving the way for wider clinical adoption.

### 4.2. Comparison with Prior Works

Inertial Measurement Units (IMUs), which are embedded in wearable and mobile devices, and Kinect have been proposed for biomechanical applications, including ROM assessment, which can be a viable and cost-effective solutions for this purpose [44,45]. Mihin [45] validated a wearable motion capture system using an IMU for assessing lower limb ROM and gait activities. Bravi et al. [44] studied the validity of a personalized wireless sensor system based on IMUs, specifically for assessing active shoulder movements, while Cubuku et al. [46] assessed the reliability and validity of the Kinect system specifically for the same joint. Also, Beshara et al. [47] utilized a combined system of inertial sensors and Kinect to assess active shoulder mobility, investigating validity, intra-rater, and inter-rater reliability. Engstrand et al. [48] proposed and validated an application to measure wrist motion using sensors embedded or connected (from wearable devices) to the smartphone. Notably, applications based on IMUs and Kinect do not feature automatic detection of KJPs, which is enabled by HPE models.

Image-based solutions are also identified in the literature. *DrGoniometer*, for example, has been evaluated in different studies [49,50,51], where it was used to assess various body segments (elbow, shoulder, knee, feet). Although the application employs computer vision techniques, none of the studies specify how the ROM measurements were implemented. However, based on the information provided in the studies, the virtual KJPs placed on the body segments are still manually positioned by the examiner. Moreover, the analysis method was limited to evaluating reliability rather than directly addressing the validity of the application. Another application is the *iPinPoint*, which was evaluated to measure the hallux valgus angles [17], pediatric forearm diaphyseal fracture angles [18], and the Cobb angle [16]. This differs from our study, as *iPinPoint* operates with X-ray images.

The rising utilization of HPE models in biomechanics-related analysis has led to an emergence of studies advocating for their application in assessing ROM, such as those by Fan et al. [23] and Sabo et al. [24]. While both studies utilized HPE models to assess ROM, notable differences between them and compared to our study are evident. Fan et al. [23] focused solely on OpenPose for assessing ROM in upper limb images, finding mean differences of less than three degrees in five movements (shoulder abduction: 0.51; shoulder elevation: 2.87; elbow flexion: 0.38; elbow extension: 0.65; wrist extension: 0.78). In contrast, Sabo et al. [24] investigated various HPE models (OpenPose, AlphaPose, Detectron, MediaPipe-Pose, MoveNet–Thunder) to analyze hypermobility in upper and lower limbs, along with spine flexion, using video analysis. Their goal was to determine the effectiveness of these models in detecting hypermobility and their potential use as screening tools. In both studies, comparative analyses were performed with a digital goniometer. Another notable difference identified in Fan et al. [23] compared with Sabo et al. and our study lies in the method of image capture. They used three commercial digital cameras strategically positioned around the participant (one in front and two on the sides), rather than using a smartphone (i.e., in a monocular view). This inherently results in a larger pool of images depicting the same movement available for evaluation, which contrasts with our study’s approach of using a single image. Additionally, data collection was conducted in a controlled environment illuminated with normal white light from LED sources.

Similar to our study, Sabo et al. [24] used a smartphone to take photos, but they were recorded using a mounted tripod, which differs from our protocol in which the device was held in the examiner’s hand. Furthermore, they did not assess the performance of all models across all movements; instead, they utilized different HPE models for evaluating different movements. They employed the AlphaPose, Detectron, MediaPipe-Pose, MoveNet–Thunder, and OpenPose HPE models for ankle, shoulder, elbow, and knee movements. For analyzing hand movements, they utilized the AlphaPose and MediaPipe-Pose HPE models. Lastly, the statistical analysis employed by Sabo et al. [24] includes only correlation analysis, showing moderate to strong correlations between measurements taken with the HPE models and those from the goniometer. For the *MoveNet Thunder*, the correlations with goniometer measurements were 0.632 and 0.893 for shoulder flexion and 0.648 and 0.804 for elbow extension. However, correlation analysis is not the most suitable method for assessing agreement between quantitative measurements. Instead, Bland–Altman analysis is considered the gold standard for evaluating method comparability [52], which was the approach utilized in our study.

### 4.3. Practical Implications

The use of HPE models can optimize the ROM assessment measures, which are critical for determining a patient’s health status at a given time, assessing the severity of motor deficits, and monitoring the progress resulting from motor rehabilitation. Users normally want near real-time results with fast, automated processing, and HPE models can reduce the processing time when compared to manual data annotation. Therefore, they serve as an alternative to the use of external markers that may interfere with an individual’s natural movement patterns, consequently hindering the analysis. HPE models offer a viable solution to address the need for a tool capable of efficiently processing parameters of interest in ROM analysis, delivering rapid results. Integrating HPE models into smartphones or other portable devices could enhance the potential for widespread adoption of ROM assessment across various clinical settings and public health applications.

While HPE models continue to advance in their application to movement analysis, caution remains imperative, particularly in the clinical evaluation of upper-limb ROM. As highlighted in this study, models may still exhibit errors in KJP detection. In clinical settings, exclusive dependence on automated KJP detection by HPE models is discouraged. New HPE solutions must incorporate standardized mechanisms that assist users in identifying improperly positioned KJPs and determining the suitability of images for the intended task. To meet this requirement, one possible solution is to incorporate active learning [53], which allows HPE models to learn from annotations provided by specialized examiners. This underscores the need for new models to undergo specific training tailored to the task of goniometry. Until such advancements are achieved, a visual inspection and manual adjustments in KJPs positioning will remain indispensable.

To use HPE models in clinical settings, health professionals must scrutinize the captured image and conduct a visual examination of the identified KJPs. If the KJPs are visibly positioned away from the joints of interest, the examiner should manually drag the detected points to the correct positions based on the examiner’s anatomical knowledge. This step is crucial for determining whether a new participant photo is necessary before proceeding with the movement analysis. While the use of HPE models expedites KJP detection, the examiner’s visual inspection remains indispensable from a clinical perspective to enhance the reliability of measurements.

### 4.4. Limitations and Future Work

In this study, we sought to gather data in a setting that closely mimics the environment typically found in motor rehabilitation clinics. Consequently, we refrained from using supplementary artificial lighting, relying solely on ambient room lights. Additionally, the examiner handheld the smartphone rather than mounting it on a tripod, and images were captured and analyzed in only a monocular view. These conditions may be considered a limitation, as the environment may have influenced the results. Using HPE models in more controlled conditions could improve their performance.

To determine if specific variables contribute to a higher frequency of outliers, further investigation is required. Future studies should consider various conditions, including complications in pose (comparing straight postures with flexed postures) and environmental and technological factors. This approach entails controlling a series of factors to identify which ones contribute most significantly to the occurrence of outliers.

Also, the comparison of the five models did not encompass the ROM for wrists and fingers. Therefore, in the future, an analysis that includes ROM measurements of these joints, as well as the lower limbs, may help to understand whether the performance pattern found here is maintained for other joint movements. In the future, we plan to broaden the range of movements assessed and incorporate HPE models specifically tailored to those movements. However, we believe that it is essential to first address the current limitations of the models, which we identified in our study, and improve their accuracy. Enhancing these models is crucial for effectively assessing complex movements, such as rotations and hand movements.

Future studies can also focus on tuning existing HPE models, when possible, or developing new models designed to make them task-specific models. This process requires the creation of specialized training datasets, including images of individuals in key ROM positions and covering a diverse range of clinical scenarios, such as different age groups, pathologies, and degrees of joint limitation. By incorporating these task-specific data, it would be possible to optimize the accuracy of models, thereby increasing its reliability and practical applicability in clinical settings.

Furthermore, future studies could evaluate the performance of additional HPE models, such as *OpenPose* and *Mediapipe*, specifically in tasks like ROM assessment. Additionally, models such as *X-Pose* and *Sapiens* could be tested as well. *X-Pose* has demonstrated effectiveness in detecting KJPs in real-world scenes [54], while *Sapiens* models exhibit strong generalization capabilities across a range of human-centric tasks [55]. However, the potential advantages of these models must also be tested in scenarios involving human body physical examinations to determine whether these benefits extend to such tasks.

Finally, in the present study, we focused on image analysis, not addressing the continuous analysis of movement data in videos. This approach was appropriate for the objectives of our study, but it limits the application of the results in contexts where real-time body dynamics are relevant. In future studies, we plan to explore both the models evaluated here and new ones for video analysis, aiming to achieve a more detailed and accurate assessment of human movement over time.

## 5. Conclusions

This study aimed to evaluate and compare the performance of different HPE models in evaluating human upper limbs ROM. Among the HPE models evaluated, *MNT16* was the one that presented the smallest error in comparison with UG. However, while this discovery offers compelling evidence that HPE models show promise in enabling quantitative ROM assessments, thereby enhancing the convenience and dynamism of the assessment process, they have not yet demonstrated sufficient accuracy for this task. Errors exceeding 10 degrees were observed, particularly in elbow flexion. Therefore, while employing HPE models in clinical settings may aid in the assessment process, it does not negate the crucial examiner’s role in meticulously scrutinizing images and adjusting KJPs to enhance measurement reliability. This highlights the need for caution among examiners when considering the automated measurement of upper limb ROM using HPE models. These findings regarding the models’ unsatisfactory performance open new avenues for research and discussions concerning their application in health assessment. While these models show promise, there are still limitations that must be addressed before they can be confidently and safely integrated into clinical practice. Therefore, the results of this study stimulate further research and discussions regarding the use of HPE models in health assessment, prompting ongoing efforts to tackle this research challenge.

## Figures and Tables

**Table 1:** Descriptive Analysis and *t*-test for shoulder movements.

			Flexion			Extension			Abduction		
			Mean ± SD	*t*-Test	*p*-Value	Mean ± SD	*t*-Test	*p*-Value	Mean ± SD	*t*-Test	*p*-Value
*MNL8Q*	Right	UG	159.54 ± 7.32	−0.037	0.98 *	38.19 ± 3.32	0.964	0.34 *	166.04 ± 7.50	−1.362	0.19 *
		Model	159.38 ± 30.44			36.57 ± 6.93			159.25 ± 24.59		
	Left	UG	159.28 ± 7.12	0.053	0.96 *	38.70 ± 3.69	1.202	0.24 *	164.66 ± 7.96	−3.596	<0.001
		Model	159.38 ± 11.28			34.35 ± 6.08			156.55 ± 37.34		
*MNL16Q*	Right	UG	159.54 ± 7.32	−1.918	0.08 *	38.19 ± 3.32	−4.331	<0.001	166.04 ± 7.50	−3.646	<0.001
		Model	162.54 ± 22.61			34.61 ± 6.09			157.42 ± 29.38		
	Left	UG	159.28 ± 7.12	−4.731	<0.001	38.70 ± 3.69	−5.734	<0.001	164.66 ± 7.96	−1.779	0.07 *
		Model	161.26 ± 9.16			33.01 ± 6.25			156.79 ± 34.54		
*MNT8Q*	Right	UG	159.54 ± 7.32	−1.960	0.26 *	38.19 ± 3.32	−2.088	0.14 *	166.04 ± 7.50	−1.932	0.07 *
		Model	157.76 ± 7.99			35.64 ± 5.18			164.03 ± 7.92		
	Left	UG	159.28 ± 7.12	−1.570	0.16 *	38.70 ± 3.69	−1.648	0.15 *	164.66 ± 7.96	0.347	0.73 *
		Model	154.22 ± 9.66			37.17 ± 5.80			165.18 ± 10.12		
*MNT16Q*	Right	UG	159.54 ± 7.25	−3.165	<0.001	38.19 ± 3.29	−4.036	<0.001	166.04 ± 7.42	0.206	0.83 *
		Model	159.89 ± 6.90			36.03 ± 4.82			163.36 ± 8.10		
	Left	UG	159.28 ± 7.05	−3.362	<0.001	38.70 ± 3.66	−3.669	<0.001	164.66 ± 7.88	−0.416	0.69 *
		Model	156.63 ± 8.02			37.50 ± 7.22			166.31 ± 7.04		
*PoseNet*	Right	UG	159.54 ± 7.32	3.188	<0.02	38.19 ± 3.32	0.209	0.83 *	166.04 ± 7.50	2.253	0.08 *
		Model	152.43 ± 28.40			40.04 ± 14.33			132.60 ± 59.35		
	Left	UG	159.28 ± 7.12	2.962	<0.001	38.70 ± 3.69	0.943	0.38 *	164.66 ± 7.96	3.375	<0.001
		Model	148.09 ± 23.72			39.24 ± 20.49			120.96 ± 62.76		

Note: * = There was no significant difference between methods (i.e., model and UG); *MNL8Q* = MoveNet Lightning INT8 quantized; *MNL16Q* = MoveNet Lightning FP16 quantized; *MNT8Q* = MoveNet Thunder INT8 quantized; *MNT16Q* = MoveNet Thunder FP16 quantized; SD = Standard Deviation.

**Table 2:** Descriptive Analysis and *t*-test for elbow movements.

			Flexion			Extension		
			Mean ± SD	*t*-Test	*p*-Value	Mean ± SD	*t*-Test	*p*-Value
*MNL8Q*	Right	UG	128.48 ± 5.78	0.282	0.76 *	6.35 ± 4.07	−1.745	0.13
		Model	108.25 ± 46.39			11.70 ± 11.58		
	Left	UG	129.30 ± 5.92	−2.150	0.04	5.72 ± 4.47	−3.408	0.03
		Model	111.46 ± 37.49			8.40 ± 7.08		
*MNL16Q*	Right	UG	128.48 ± 5.78	−3.201	<0.001	6.35 ± 4.07	0.900	0.41
		Model	103.39 ± 43.91			6.56 ± 4.07		
	Left	UG	129.30 ± 5.92	−1.129	0.27 *	5.72 ± 4.47	0.178	0.87
		Model	110.36 ± 36.56			6.55 ± 4.37		
*MNT8Q*	Right	UG	128.48 ± 5.78	−2.589	0.02	6.35 ± 4.07	−4.113	<0.001
		Model	129.21 ± 25.87			10.98 ± 13.60		
	Left	UG	129.30 ± 5.92	1.429	0.16	5.72 ± 4.47	−5.036	<0.001
		Model	127.80 ± 26.83			9.66 ± 8.61		
*MNT16Q*	Right	UG	128.48 ± 5.72	−0.541	0.59 *	6.35 ± 4.02	15.182	<0.001
		Model	126.43 ± 27.84			9.18 ± 6.19		
	Left	UG	129.30 ± 5.86	−1.083	0.30 *	5.72 ± 4.43	9.639	<0.001
		Model	125.55 ± 25.89			7.98 ± 7.91		
*PoseNet*	Right	UG	128.48 ± 5.78	3.394	<0.001	6.35 ± 4.07	0.540	0.59
		Model	143.81 ± 6.63			6.78 ± 5.12		
	Left	UG	129.30 ± 5.92	1.941	0.07 *	5.72 ± 4.47	3.264	0.02
		Model	140.29 ± 7.27			21.89 ± 38.10		

Note: * = There was no significant difference between methods (i.e., model and UG); *MNL8Q* = MoveNet Lightning INT8 quantized; *MNL16Q* = MoveNet Lightning FP16 quantized; *MNT8Q* = MoveNet Thunder INT8 quantized; *MNT16Q* = MoveNet Thunder FP16 quantized; SD = Standard Deviation.

**Table 3:** ROM errors and bias of HPE models comparing with UG for shoulder movements.

		Flexion		Extension		Abduction	
		Right	Left	Right	Left	Right	Left
*MNL8Q*	BIAS ± SD	−0.16 ± 30.96	0.10 ± 13.65	−1.63 ± 6.12	−4.35 ± 6.64	−6.78 ± 24.96	−8.11 ± 37.26
	LOA	−9.75 to 7.65	−3.62 to 4.63	−3.33 to 0.01	−6.16 to −2.57	−15.49 to 0.95	−19.35 to 1.32
	RMSE	30.67	13.52	6.28	7.88	25.64	37.79
*MNL16Q*	BIAS ± SD	2.99 ± 22.40	1.98 ± 11.92	−3.58 ± 5.96	−5.69 ± 7.15	−8.62 ± 29.77	−7.87 ± 34.44
	LOA	−5.35 to 8.28	−1.45 to 5.58	−5.17 to −1.90	−7.73 to −3.66	−18.61 to 1.71	−20.72 to 1.63
	RMSE	22.39	11.97	6.90	9.08	30.72	35.01
*MNT8Q*	BIAS ± SD	−1.78 ± 9.42	−5.05 ± 10.13	−2.56 ± 5.06	−1.53 ± 6.21	−2.01 ± 7.51	0.52 ± 10.79
	LOA	−4.36 to 1.14	−7.75 to −2.16	−4.05 to −1.16	−3.11 to 0.04	−4.28 to 0.22	−2.40 to 3.46
	RMSE	9.49	11.24	5.63	6.34	7.70	10.70
*MNT16Q*	BIAS ± SD	0.35 ± 9.16	−2.62 ± 8.93	−2.16 ± 4.89	−1.20 ± 7.74	−2.68 ± 7.55	1.65 ± 8.38
	LOA	−2.12 to 2.67	−4.97 to −0.13	−3.54 to −0.70	−3.41 to 1.15	−4.96 to −0.43	−0.27 to 3.59
	RMSE	8.99	9.28	5.33	7.77	7.94	8.48
*PoseNet*	BIAS ± SD	−7.11 ± 29.38	−11.18 ± 23.67	1.84 ± 14.79	0.53 ± 21.54	−33.44 ± 58.64	−43.70 ± 62.58
	LOA	−17.77 to 0.01	−19.49 to −5.10	−1.73 to 6.33	−4.76 to 6.25	−49.76 to −17.96	−61.83 to −25.16
	RMSE	29.96	25.98	14.76	21.34	67.01	75.84

Note: *MNL8Q* = MoveNet Lightning INT8 quantized; *MNL16Q* = MoveNet Lightning FP16 quantized; *MNT8Q* = MoveNet Thunder INT8 quantized; *MNT16Q* = MoveNet Thunder FP16 quantized; SD = Standard Deviation.

**Table 4:** ROM errors and bias of HPE models comparing with UG for elbow movements.

		Flexion		Extension	
		Right	Left	Right	Left
*MNL8Q*	BIAS ± SD	−20.23 ± 46.09	−17.84 ± 38.27	5.34 ± 12.09	2.68 ± 6.52
	LOA	−33.47 to −7.06	−31.34 to −7.26	2.48 to 8.66	0.84 to 4.55
	RMSE	49.94	41.89	13.12	6.99
*MNL16Q*	BIAS ± SD	−25.08 ± 44.81	−18.94 ± 37.22	0.21 ± 7.17	0.83 ± 6.32
	LOA	−38.52 to −13.29	−28.43 to −10.67	−1.52 to 2.08	−1.01 to 2.49
	RMSE	50.98	41.45	7.11	6.32
*MNT8Q*	BIAS ± SD	0.73 ± 25.68	−1.49 ± 25.99	4.63 ± 14.83	3.93 ± 8.41
	LOA	−8.34 to 7.81	−10.34 to 4.78	1.52 to 8.24	2.00 to 5.98
	RMSE	25.44	25.78	15.40	9.21
*MNT16Q*	BIAS ± SD	−2.04 ± 27.57	−3.75 ± 25.23	2.79 ± 6.01	2.26 ± 8.47
	LOA	−9.99 to 4.64	−11.85 to 3.07	1.24 to 4.40	−0.17 to 4.76
	RMSE	27.39	25.27	6.66	8.74
*PoseNet*	BIAS ± SD	15.33 ± 7.28	10.99 ± 8.22	0.42 ± 5.60	16.17 ± 36.72
	LOA	13.07 to 17.32	8.60 to 13.55	−1.12 to 1.98	8.21 to 24.07
	RMSE	16.94	13.68	5.89	39.91

Note: *MNL8Q* = MoveNet Lightning INT8 quantized; *MNL16Q* = MoveNet Lightning FP16 quantized; *MNT8Q* = MoveNet Thunder INT8 quantized; *MNT16Q* = MoveNet Thunder FP16 quantized; SD = Standard Deviation.

## Data Availability

The raw data supporting the conclusions of this article will be made available by the authors on request.

## Supplementary Materials

The following supporting information can be downloaded at: https://www.mdpi.com/article/10.3390/s24247983/s1.

## Abbreviations

The following abbreviations are used in this manuscript:

CV	Computer vision
HPE	Human Pose Estimation
KJP	Key Joint Points
IMUs	Inertial Measurement Units
LOA	Limits of Agreements
PT-BR	Brazilian Portuguese
RMSE	Root Mean Square Errors
ROM	Range of Motion
UG	Universal Goniometer
VG	Virtual Goniometer
