# Supplementary material for: Validity Analysis of Monocular Human Pose Estimation Models Interfaced with a Mobile Application for Assessing Upper Limb Range of Motion
Source: Sensors (Basel). 2024 Dec 14;24(24):7983. doi: 10.3390/s24247983 (PMC11679233; doi:10.3390/s24247983)
Supplement: Supplementary file 1 [file sensors-24-07983-s001.zip › sensors-3351137-supplementary.pdf]

Supplementary Material S1

Bland Altman Plot for MoveNet Lightning INT8 quantized

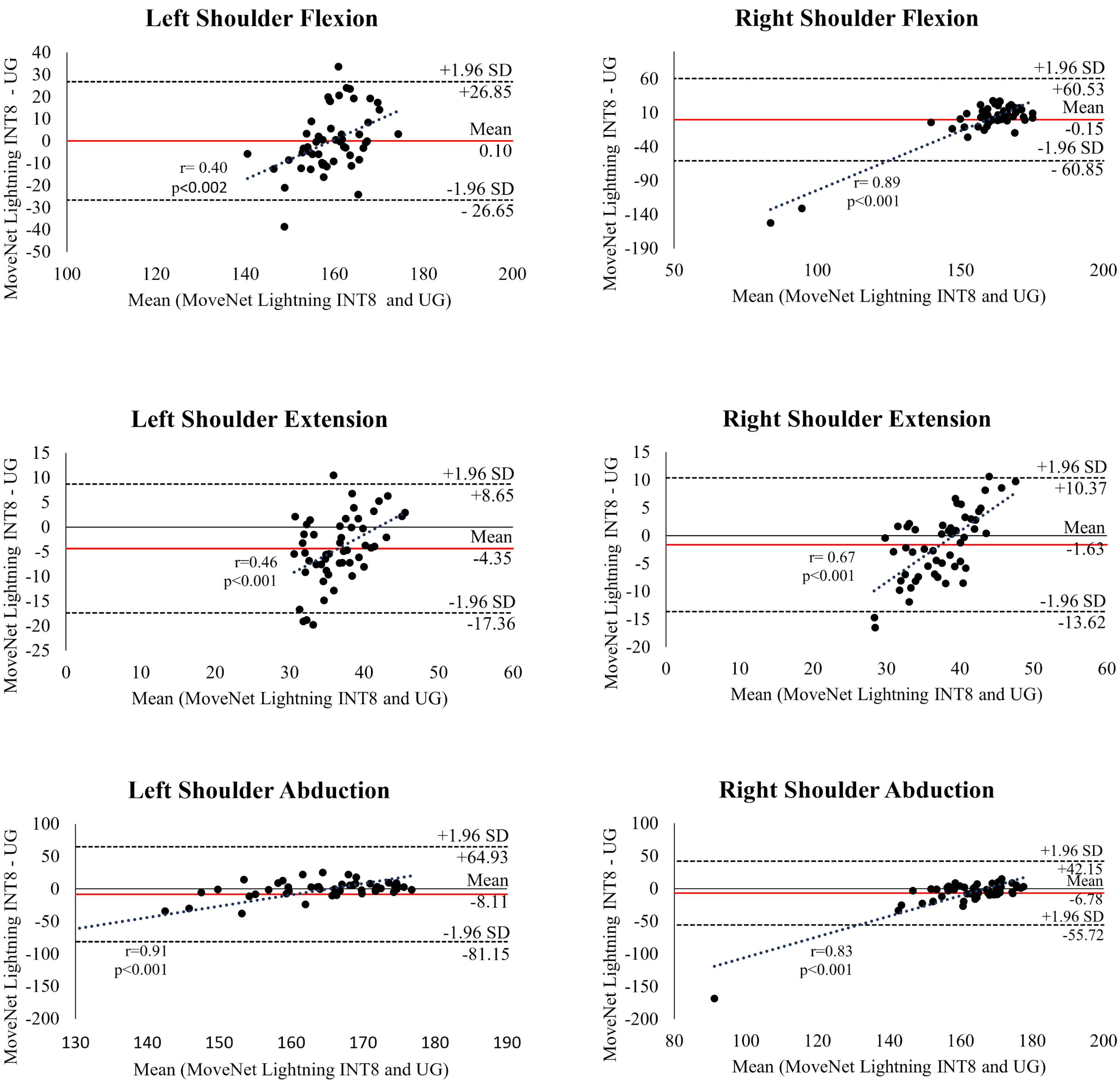

Bland Altman Plot showing the level of agreement between the MoveNet Lightning INT8 quantized and the UG when assessing shoulders. The centered red line shows mean difference, and the two outer dotted lines represent 95% confidence interval.

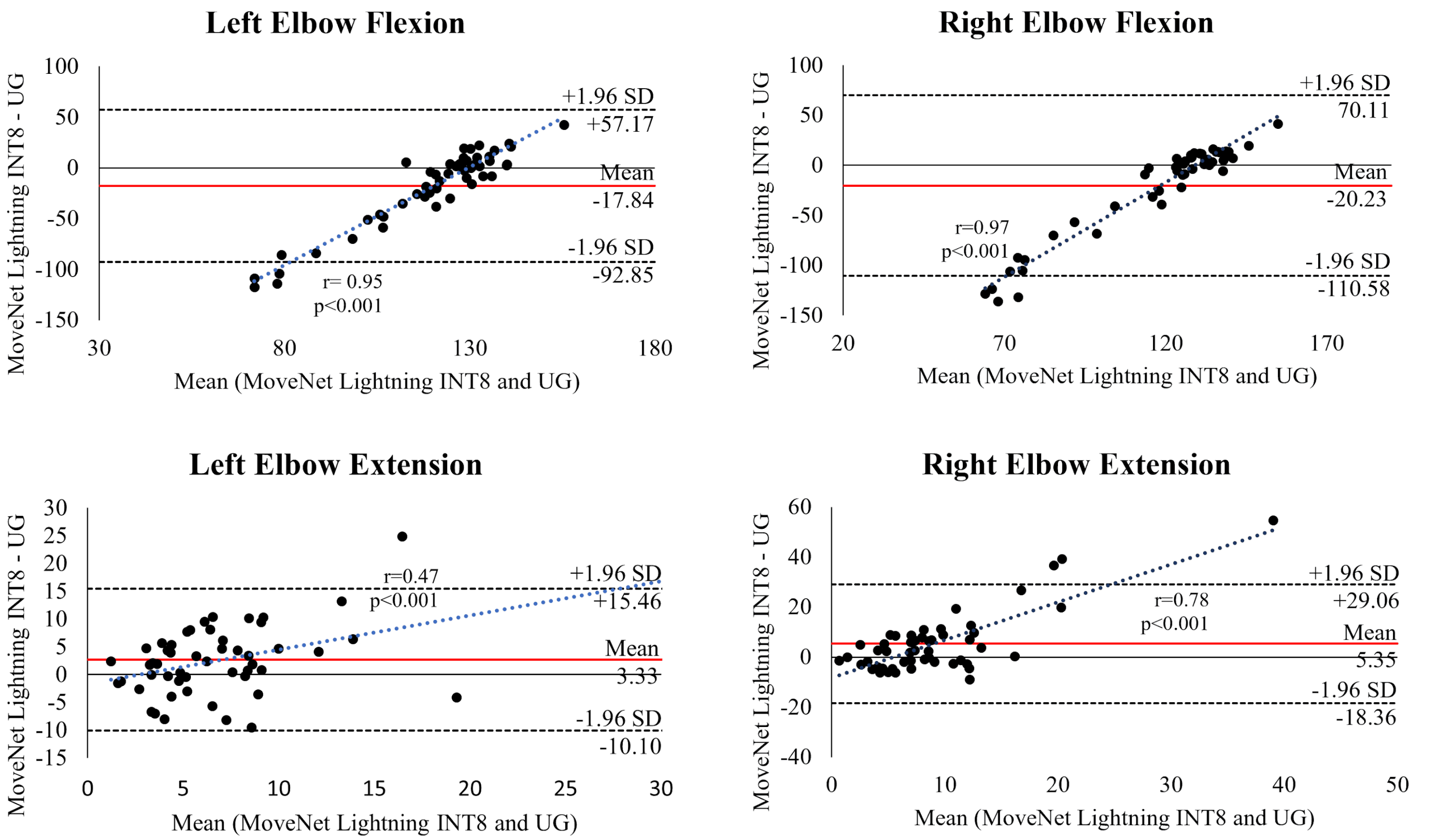

Bland Altman Plot showing the level of agreement between the MoveNet Lightning INT8 quantized and the UG when assessing elbows. The centered red line shows mean difference, and the two outer dotted lines represent 95% confidence interval.

Bland Altman Plot for MoveNet Lightning FP16 quantized

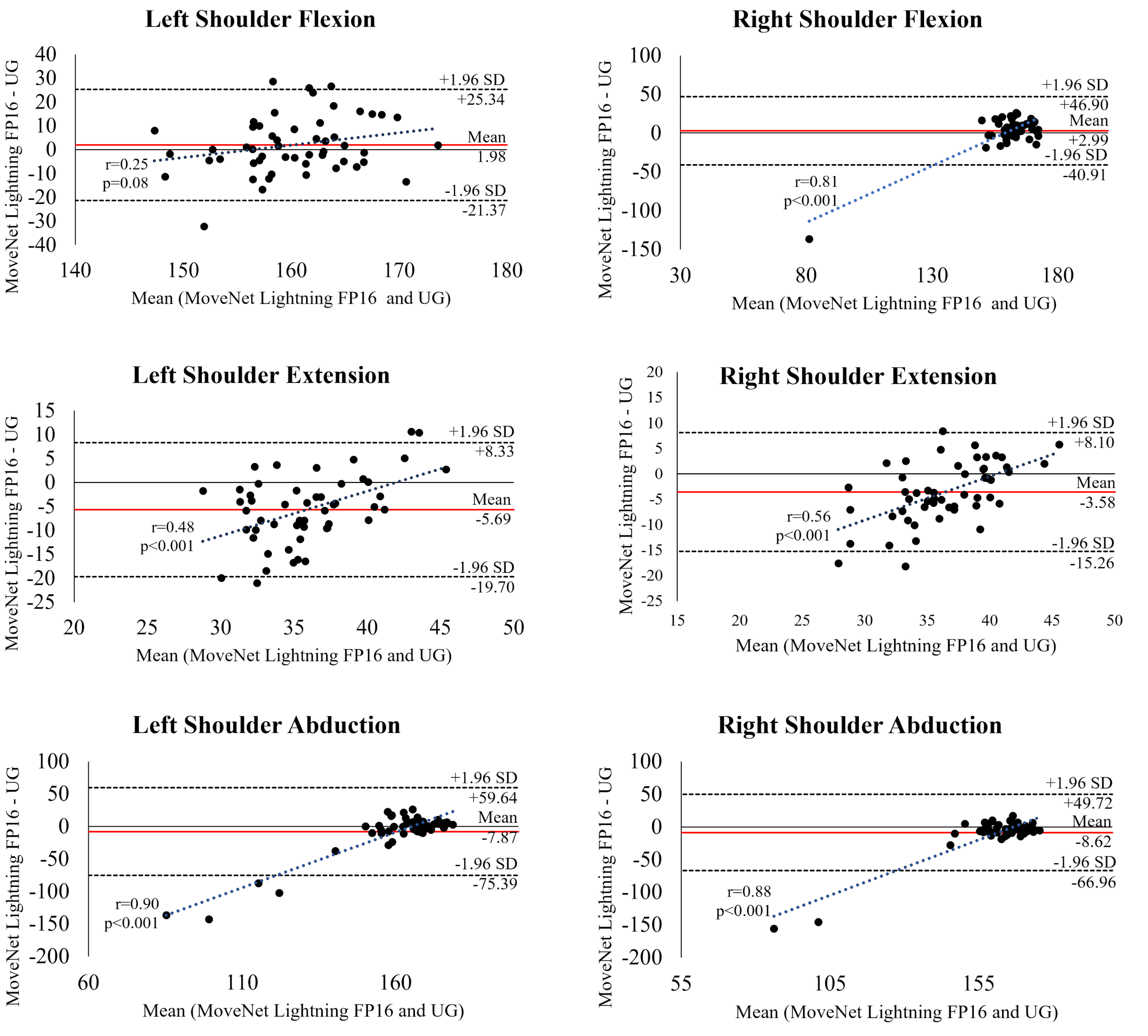

Bland Altman Plot showing the level of agreement between the MoveNet Lightning FP16 quantized and the UG when assessing shoulders. The centered red line shows mean difference, and the two outer dotted lines represent 95% confidence interval.

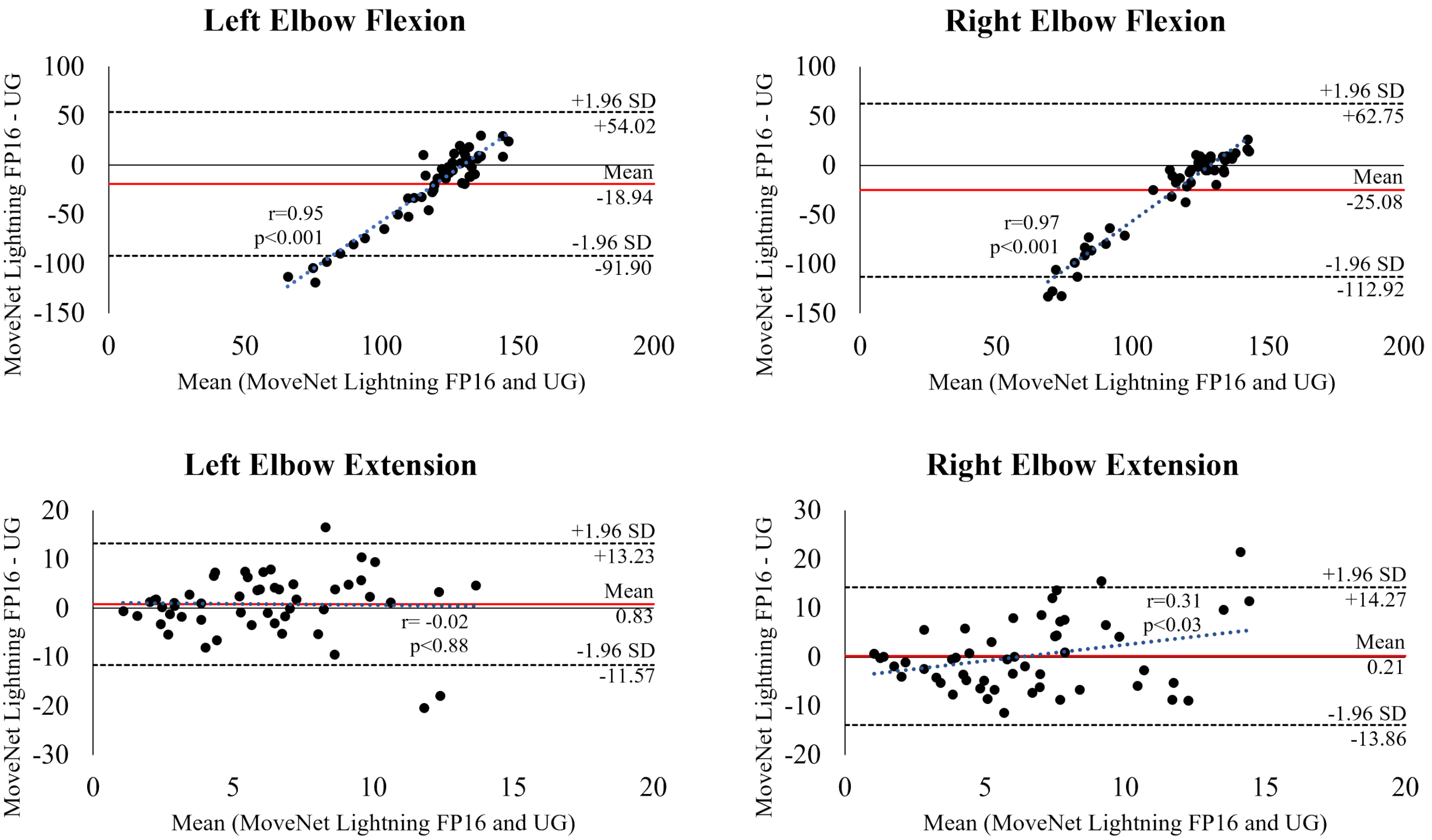

Bland Altman Plot showing the level of agreement between the MoveNet Lightning FP16 quantized and the UG when assessing elbows. The centered red line shows mean difference, and the two outer dotted lines represent 95% confidence interval.

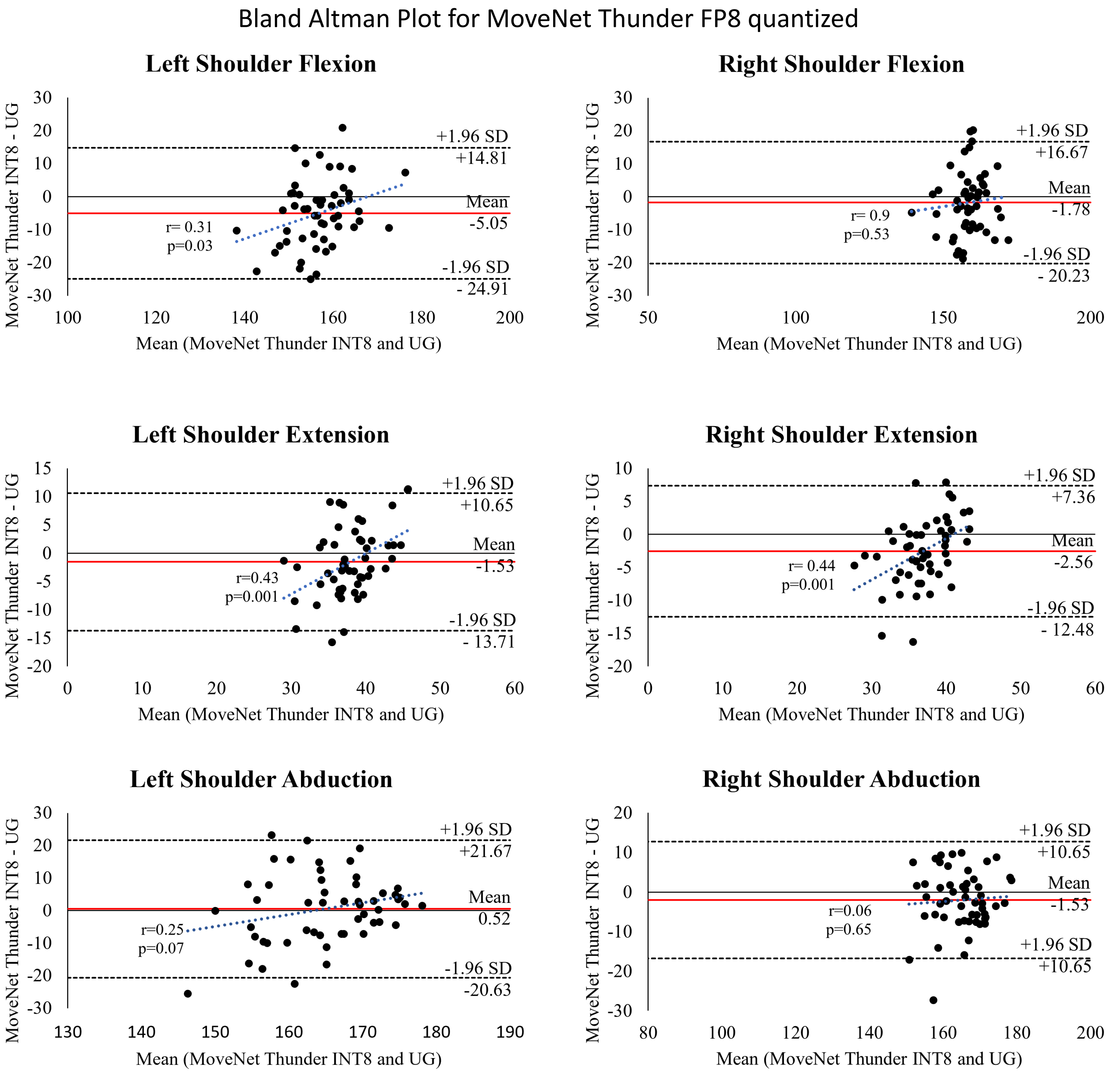

Bland Altman Plot showing the level of agreement between the MoveNet Thunder FP8 quantized and the UG when assessing shoulders. The centered red line shows mean difference, and the two outer dotted lines represent 95% confidence interval.

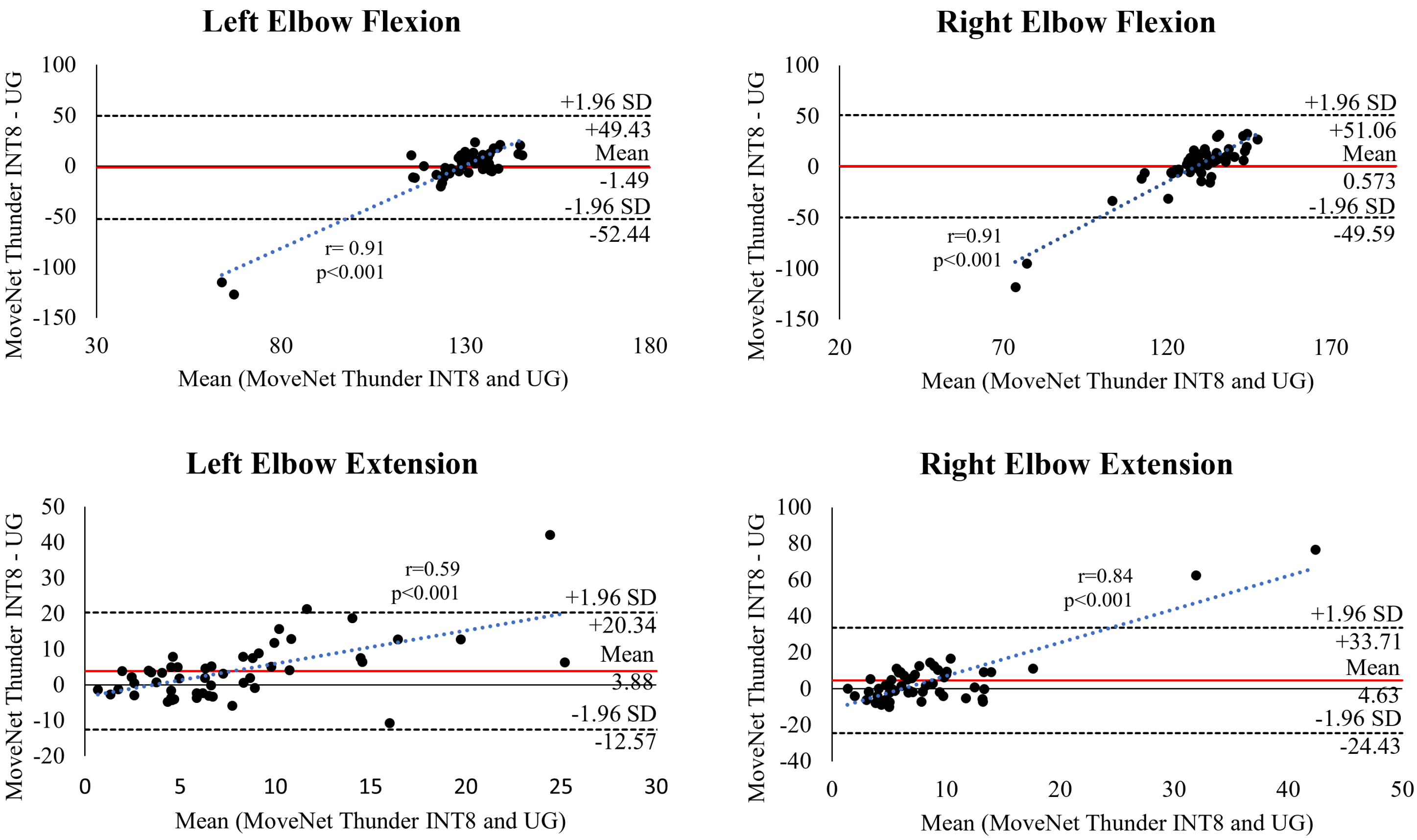

Bland Altman Plot showing the level of agreement between the MoveNet Thunder FP8 quantized and the UG when assessing elbows. The centered red line shows mean difference, and the two outer dotted lines represent 95% confidence interval.

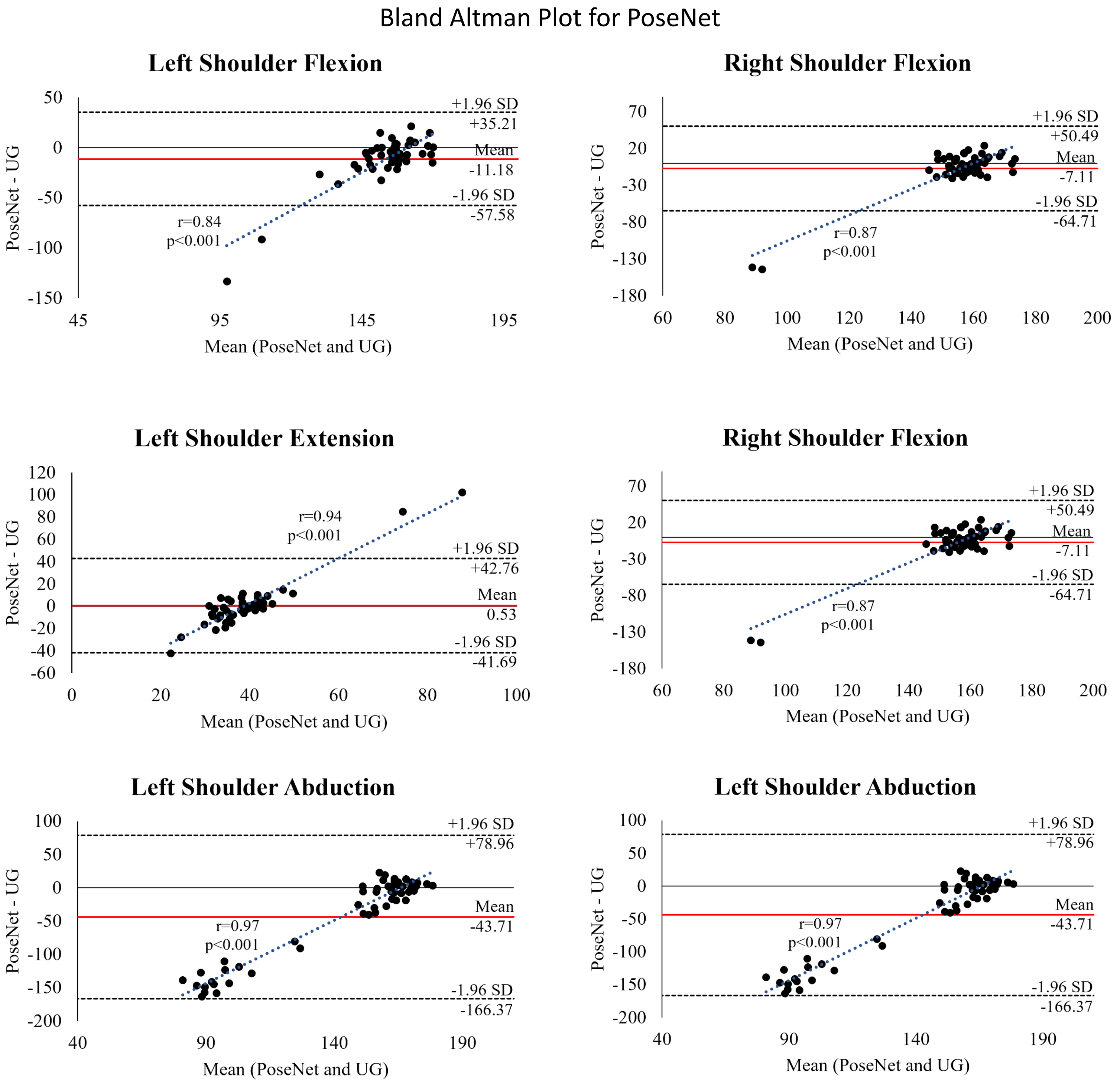

Bland Altman Plot showing the level of agreement between the PoseNet and the UG when assessing shoulders. The centered red line shows mean difference, and the two outer dotted lines represent 95% confidence interval.

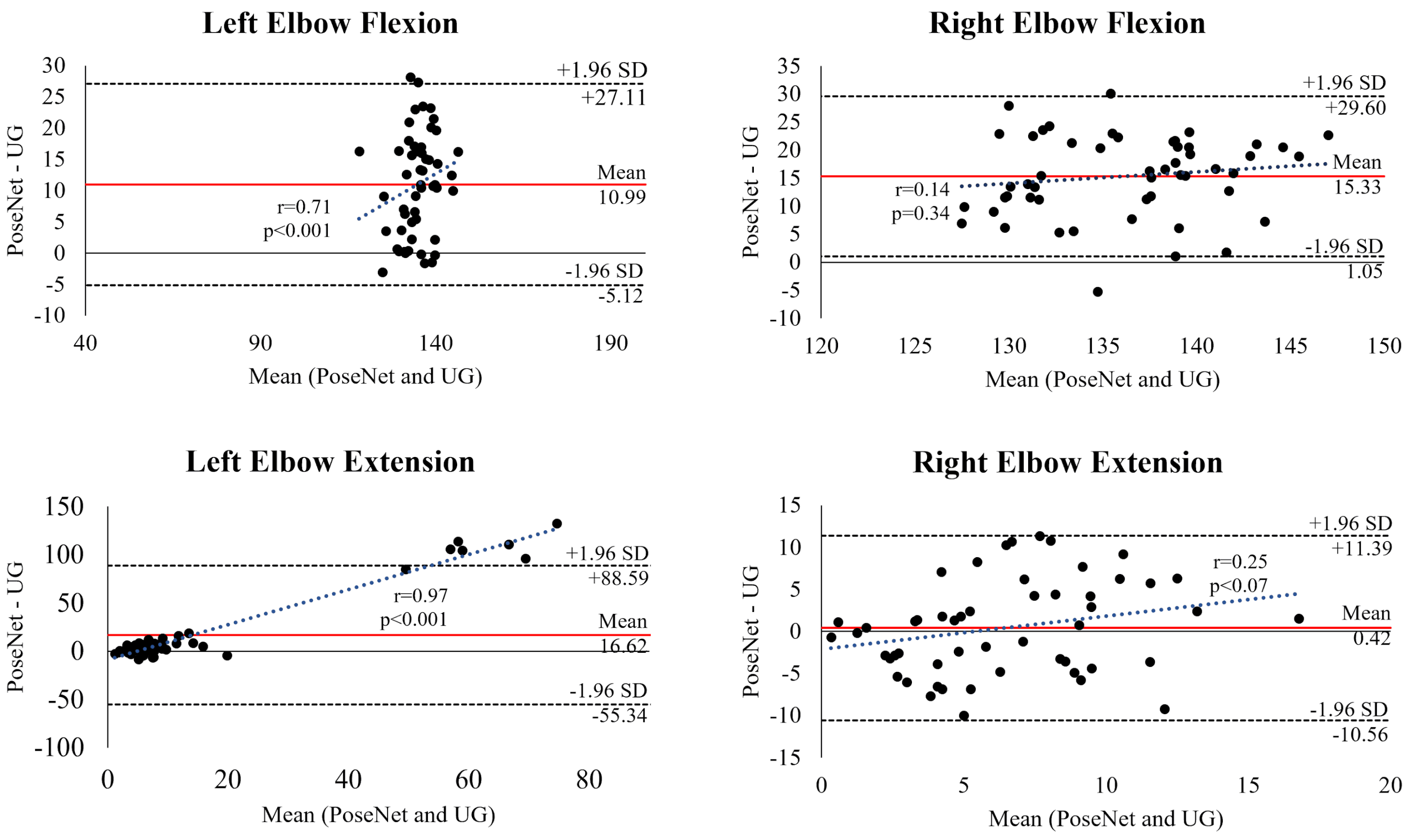

Bland Altman Plot showing the level of agreement between the PoseNet and the UG when assessing elbows. The centered red line shows mean difference, and the two outer dotted lines represent 95% confidence interval.
